# Improving public policies for people with ADHD, Autism and AuDHD – Leveraging administrative data to understand current population level trends in Canada and learning from policies internationally

**DOI:** 10.1192/j.eurpsy.2026.10751

**Published:** 2026-07-31

**Authors:** C. G. Roth, J. Zwicker, K. Fyie

**Affiliations:** School of Public Policy, University of Calgary, Calgary, Canada

## Abstract

**Introduction:**

People with ADHD and autism experience a wide range of adverse health, education and socio-economic outcomes. There are barriers and differences between the two diagnoses in accessing health and educational services and disability supports for children/youth.

**Objectives:**

1. What are the recent prevalence rates, outcomes, and service uses of children/youth with ADHD, autism and AuDHD (dual diagnosis), and

2. How do policies internationally address ADHD and autism supports.

**Methods:**

**1.** Statistical Analysis: The study uses recent administrative data from the Province of British Columbia (BC) in Canada to describe prevalence rates, relationships with selected health, educational and socio-economic outcomes, as well as current access to supports and services received by children/youth (0-18 years old). Descriptive statistics and regression modelling is used (stratified based on social and demographic factors). Exposure: ADHD and/or autism diagnosis. Outcome variables: Mental health disorders, high school graduation, sexually transmitted diseases and criminal involvement. Potential modifiers: access to health care services (e.g. age of diagnosis, pharmacological treatment), public disability support use, education support use.

**2.** Policy Scan: Analysis of Autism or ADHD targeted policies in English speaking OECD countries.

**Results:**

**Administrative data analysis:**

**Table 1.** Key Statistics on ADHD, Autism, and AuDHD among Children and Youth (2023) Table 1. Long description.

Measure	ADHD	Autism
**Prevalence**	8.6% (m=5.7%, f= 2.92%)	3.3% (m=2.4%, f=0.9%)
**Increase in diagnoses (10 years)**	+103%	+252%
**Comorbid mental health condition**	36% (m=32%, f=43%)	29% (m=27%, f=35%)
**Comorbid neurodevelopmental condition**	tbc	63% (m=64%, f=61%)
**ADHD Prescription drug use**	39% in 2014, 45% in 2023	–
**Educational supports received**	<20%	<50%

**Policy Scan Results:** More policies exist related to autism. Policies rarely consider dual diagnosis. Most policies focus on health or education. Recent trend towards more cross-sectoral policy efforts.

**Image 1:**
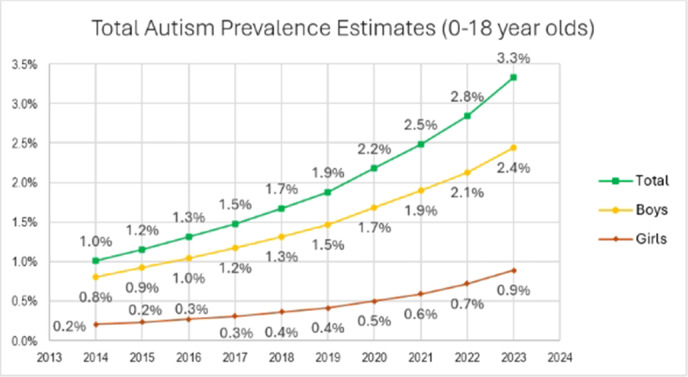
Image 1: Long description.

**Image 2:**
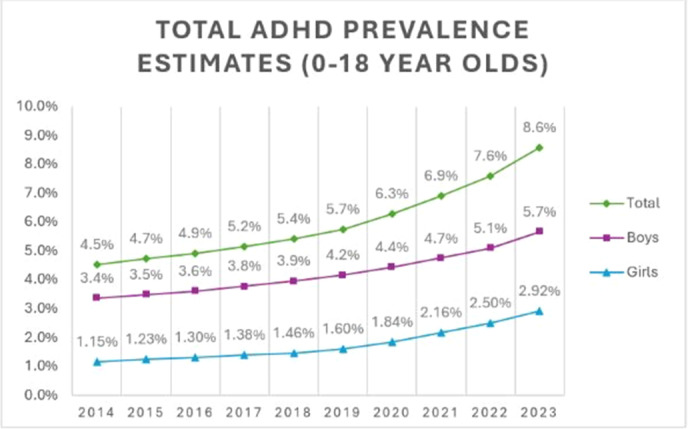
Image 2: Long description.

**Image 3:**
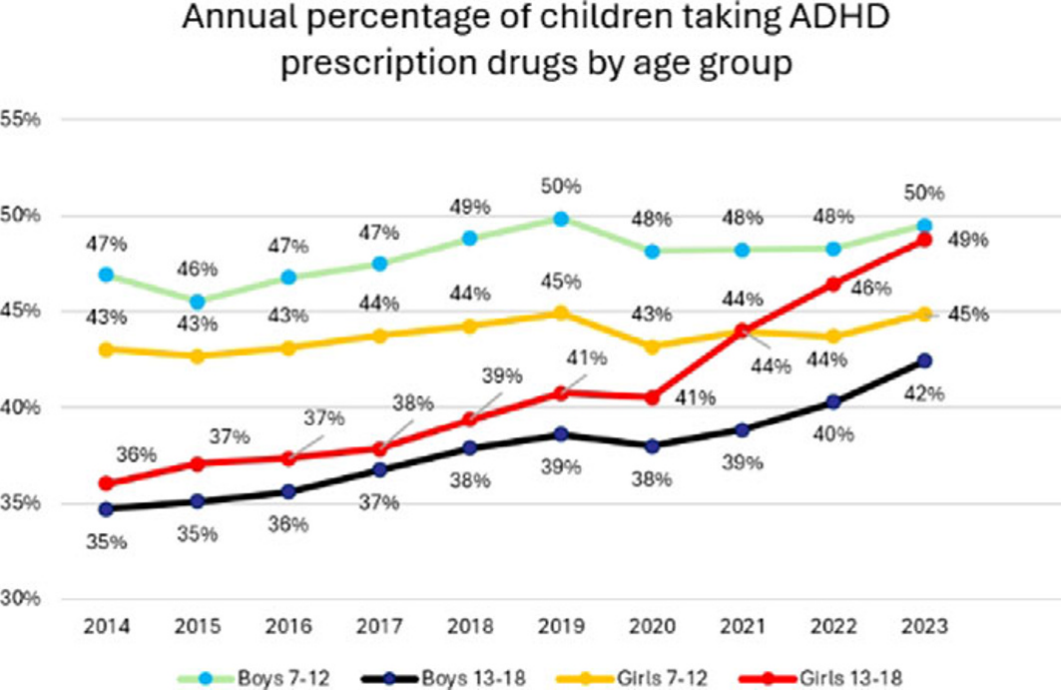
Image 3: Long description.

**Conclusions:**

Findings show increased rates of ADHD, autism and proportionately higher rates of dual diagnoses. There are global gaps in policy efforts to address the rapid increase of diagnoses. Recommendations can improve the design and access of health, educational and disability support services in Canada and internationally.

**Disclaimer:**

All inferences, opinions, and conclusions drawn in these materials are those of the authors. They do not reflect the opinions or policies of the provider(s) of the data upon which they are based.

**Disclosure of Interest:**

None Declared

## Long descriptions

### Table 1. Long description

The table consists of three columns: Measure, A D H D, and Autism.

* Prevalence: A D H D is 8.6% (males 5.7%, females 2.92%); Autism is 3.3% (males 2.4%, females 0.9%).

* Increase in diagnoses over 10 years: A D H D increased by 103%; Autism increased by 252%.

* Comorbid mental health condition: A D H D is 36% (males 32%, females 43%); Autism is 29% (males 27%, females 35%).

* Comorbid neurodevelopmental condition: A D H D is listed as t b c; Autism is 63% (males 64%, females 61%).

* A D H D Prescription drug use: A D H D was 39% in 2014 and 45% in 2023; no data provided for Autism.

* Educational supports received: Less than 20% for A D H D; less than 50% for Autism.

Navigate back to Table 1..

### Image 1: Long description

The x-axis represents years from 2013 to 2024, and the y-axis represents prevalence percentage from 0.0 percent to 3.5 percent. Three lines track the data from 2014 to 2023.

* The top line (green squares) represents the Total prevalence. It starts at 1.0 percent in 2014 and shows a consistent upward curve, reaching 1.5 percent in 2017, 2.2 percent in 2020, and peaking at 3.3 percent in 2023.

* The middle line (yellow circles) represents Boys. It follows a similar trajectory, starting at 0.8 percent in 2014, rising to 1.2 percent in 2017, 1.7 percent in 2020, and reaching 2.4 percent in 2023.

* The bottom line (orange diamonds) represents Girls. It shows a much lower but also increasing prevalence, starting at 0.2 percent in 2014, reaching 0.3 percent in 2017, 0.5 percent in 2020, and ending at 0.9 percent in 2023.

The gap between the prevalence for boys and girls widens over time as the overall rates increase.

Navigate back to Image 1:.

### Image 2: Long description

The y-axis represents prevalence percentage from 0.0 percent to 10.0 percent in 1.0 percent increments. The x-axis represents years from 2014 to 2023. A legend to the right identifies three lines.

* Total (green line with diamond markers): Shows a non-linear increase starting at 4.5 percent in 2014, rising steadily to 5.7 percent in 2019, and then accelerating to 8.6 percent by 2023.

* Boys (purple line with square markers): Shows a steady upward trend starting at 3.4 percent in 2014, reaching 4.2 percent in 2019, and ending at 5.7 percent in 2023.

* Girls (blue line with triangle markers): Shows the lowest prevalence but the most consistent upward curve, starting at 1.15 percent in 2014, reaching 1.60 percent in 2019, and rising to 2.92 percent in 2023.

All three demographics show a continuous year-over-year increase throughout the decade.

Navigate back to Image 2:.

### Image 3: Long description

The x-axis represents years from 2014 to 2023. The y-axis represents the percentage of children, ranging from 30 percent to 55 percent. The graph tracks four demographic groups.

* Boys 7 to 12 (light green line with blue dots): Starts at 47 percent in 2014, fluctuates slightly, peaks at 50 percent in 2019 and 2023, and ends at 50 percent.

* Girls 13 to 18 (red line): Shows a steady upward trend from 36 percent in 2014 to 49 percent in 2023, with a notable sharp increase after 2020.

* Girls 7 to 12 (yellow line): Remains relatively stable, starting at 43 percent in 2014, dipping slightly in 2015 and 2020, and ending at 45 percent in 2023.

* Boys 13 to 18 (black line): Shows a gradual increase from 35 percent in 2014 to 42 percent in 2023.

Overall, all groups show an increase in medication use over the decade, with the most significant growth occurring in the Girls 13 to 18 age group.

Navigate back to Image 3:.

